# Integrated Bioinformatical Analysis Identifies GIMAP4 as an Immune-Related Prognostic Biomarker Associated With Remodeling in Cervical Cancer Tumor Microenvironment

**DOI:** 10.3389/fcell.2021.637400

**Published:** 2021-01-21

**Authors:** Fangfang Xu, Jiacheng Shen, Shaohua Xu

**Affiliations:** Department of Gynecology, Shanghai First Maternity and Infant Hospital, School of Life Sciences and Technology, Tongji University, Shanghai, China

**Keywords:** GIMAP4, tumor microenvironment, somatic mutation, cervical cancer, ESTIMATE, CIBERSORT

## Abstract

Tumor microenvironment (TME) is emerging as an essential part of cervical cancer (CC) tumorigenesis and development, becoming a hotspot of research these years. However, comprehending the specific composition of TME is still facing enormous challenges, especially the immune and stromal components. In this study, we downloaded the RNA-seq profiles and somatic mutation data of 309 CC cases from The Cancer Genome Atlas (TCGA) database, which were analyzed by integrative bioinformatical methods. Initially, ESTIMATE computational method was employed to calculate the amount of immune and stromal components. Then, based on the high- and low-immunity cohorts, we recognized the differentially expressed genes (DEGs) as well as the differentially mutated genes (DMGs). Additionally, we conducted an intersection analysis of DEGs and DMGs, ultimately determining an immune-related prognostic signature, GTPase, IMAP Family Member 4 (GIMAP4). Moreover, sequential analyses demonstrated that GIMAP4 was a protective factor in CC, positively correlated with the overall survival (OS) and negatively with distant metastasis. Besides, we utilized the Gene Set Enrichment Analysis (GSEA) to explore the enrichment-pathways in high and low-expression cohorts of GIMAP4. The results indicated that the genes of the high-expression cohort had a high enrichment in immune-related biological processes and metabolic activities in the low one. Furthermore, CIBERSORT analysis was applied to evaluate the proportion of tumor-infiltrating immune cells (TICs), illustrating that several activated TICs were strongly associated with GIMAP4 expression, which suggested that GIMAP4 had the potential to be an indicator for the immune state in TME of CC. Hence, GIMAP4 contributed to predicting the CC patients’ clinical outcomes, such as survival rate, distant metastasis and immunotherapy response. Moreover, GIMAP4 could serve as a promising biomarker for TME remodeling, suggesting the possible underlying mechanisms of tumorigenesis and CC progression, which may provide different therapeutic perceptions of CC, and therefore improve treatment.

## Introduction

Cervical cancer (CC) is the fourth malignancy worldwide in the female reproductive system, representing a major global health challenge. There are more than 500,000 women diagnosed with CC and over 300,000 deaths worldwide each year, of which about 84 percent occurs in economically underdeveloped areas, and the proportion is expected to grow to 98 percent by 2030; additionally, the overall prognosis remains poor for women with metastatic or recurrent disease (Cohen et al., 2019; Pesola and Sasieni, 2019). The increasing CC morbidity is closely linked to human papillomavirus (HPV) infection, chronic cervical lesions, genetic modification, and many other factors. The currently known genetic alterations associated with CC involve the ErbB-3 (Di et al., 2015; Cancer Genome Atlas Research Network et al., 2017), epidermal growth factor receptor (EGFR) (Wei et al., 2018), Serine/Threonine Kinase 11 (STK11) (Hirose et al., 2020), transforming growth factor-beta receptor 2 (TGFBR2) (Cai et al., 2018), phosphatase and tensin homolog (PTEN) (Nero et al., 2019), etc. Nevertheless, the underlying mechanisms of CC carcinogenesis and progression still remain elusive so far. The treatments for CC have remained unchanged for several decades, such as surgical treatment, cytotoxic chemotherapy, and radiotherapy, etc. These therapy approaches can hardly prevent the metastasis and recurrence in CC patients; thus, new therapeutic strategies are urgently required to improve the poor prognosis. Despite checkpoint inhibitor-based immunotherapy is emerging as a novel therapeutic approach and has achieved enormous success in various kinds of cancer currently, the response of CC patients remains low, which restricts the development and application of immunotherapy in CC (Di et al., 2015; Herbst et al., 2016; Schachter et al., 2017). Besides, although continuous development in these conventional and newly generated therapeutic methods have been achieved and are gradually applied clinically for CC these years, the 5-year disease-free survival (DFS) rates are merely 45% for CC patients with advanced-stage (Chopra et al., 2018). Therefore, there is an impendency to explore and identify the molecular aberrations to investigate the carcinogenesis mechanisms and therapeutic strategies of CC.

These years, the tumor environment (TME) is attracting growing attention and becoming a research hotspot since its complicated composition and fluctuating status played a vital role in tumorigenesis and cancer progression (Zhou et al., 2018). Besides, the increasing evidence indicates that tumor-infiltrating immune cells (TICs) and stromal components have a tight connection to the development of CC (Zhou et al., 2017; De Nola et al., 2019). Among them, the immune component seems to contribute more to immunotherapy-response of CC, including the T cells, macrophages, and neutrophils (Langers et al., 2014; Krishnan et al., 2018; Chen X. J. et al., 2019; Ohno et al., 2020). Many researchers have currently investigated the correlation of the prognosis in CC patients with the critical immunological biomarkers (De Jaeghere et al., 2020; Yuan et al., 2020; Zhao et al., 2020). These studies showed significant heterogeneity of the immune component and immune response in CC patients, which might play a decisive role in the ultimate clinical outcomes of patients. However, it is still challenging to understand and clarify the biological characteristics and effects of TME in CC patients. Hence, carrying out a detailed analysis of the genetic layer to properly illustrate the dynamic transition of TME is becoming more and more indispensable, which might help demonstrate the underlying mechanisms of carcinogenesis and progression in CC patients.

We downloaded the transcriptome RNA-seq profiles and somatic mutation information from The Cancer Genome Atlas (TCGA) database for this study, aiming to discern some prognostic immune-related biomarkers in TME of CC using integrative bioinformatics methods. Firstly, the ESTIMATE computational method was applied to calculate the respective proportion of immune and stromal ingredients of CC cases, and further analyses were conducted based on the high- and low-immunity groups since we found that the immune component was more likely to forecast the overall survival (OS) rate of CC patients. Secondly, the somatic mutation data was also analyzed by comparing the high- and low-immunity groups, revealing some significant differences in these two groups’ genetic mutation level. Thirdly, 1,067 differentially expressed genes (DEGs) and 32 differentially mutated genes (DMGs) were recognized using the above analyses. The intersection analysis of DEGs and DMGs revealed an immune-related predictive biomarker, GTPase, IMAP Family Member 4 (GIMAP4). GIMAP4 was reported to be closely connected with the immune biological process of T helper (Th) cell differentiation by regulating some specific cytokines like interleukin-4 (IL-4), interferon-γ (INF-γ), and interleukin-12 (IL-12) (Filen et al., 2009; Filen and Lahesmaa, 2010; Heinonen et al., 2015), demonstrating that GIMAP4 might play an essential part in TME. Therefore, the immune-related biological characteristics of GIMAP4 were further analyzed using Gene Set Enrichment Analysis (GSEA) and CIBERSORT. Finally, the correlation of GIMAP4 with common inhibitory checkpoint molecules (I) was analyzed to evaluate the immunotherapy response targeting immune checkpoint inhibitors (ICIs) of CC patients. The present results demonstrated GIMAP4 as an immune-related predictive biomarker, suggesting it might occupy an important place in different TME status of CC patients. Here we embarked from DEGs and DMGs generated by comparison between the immune component in CC cases and indicated that the GIMAP4 might serve as a potential immune-related predictive biomarker and an indicator for remodeling TME in CC, suggesting the possible underlying mechanisms of the tumorigenesis and progression of CC, and therefore improve treatment.

## Materials and Methods

### Raw Data

Transcriptome profiles and somatic mutation information of 309 CC cases (normal samples, 3 cases; tumor samples, 306 cases) were retrieved from the TCGA database^1^ and the corresponding clinical data from cBioportal^2^.

### Estimation for ImmuneScore, StromalScore, and ESTIMATEScore

ImmuneScore (proportion of immune ingredient), StromalScore (proportion of stromal ingredient), and ESTIMATEScore (sum of the above two scores) of each CC sample were calculated using ESTIMATE package by R software (version: 3.6.3) (Yoshihara et al., 2013), which means that the higher score represents the more considerable amount of the corresponding component (immune, stromal, and tumor purity) in TME.

### Survival Analysis

Survival analysis was carried out using both survminer and survival packages by R software. We screened out 232 tumor samples out of 309 CC cases considering the following conditions: I. Remove samples whose survival time shorter than 1 month, II. Remove normal samples, III. Remove samples with uncompleted clinical information. Survival curve was drawn through Kaplan–Meier method. The statistical significance was tested via log-rank with the significant threshold of *p*-value set as 0.05.

### Correlation Analysis of Scores With Clinicopathological Characteristics

Package ggpubr was loaded to perform the correlation analysis of each score with clinicopathological characteristics. The statistical significance was tested by Wilcoxon rank sum test or Kruskal-Wallis rank sum test.

### Somatic Mutation Analysis and Identification of DMGs in High- and Low-Immunity Cohorts Regarding ImmuneScore

Somatic mutation information of CC was retrieved from the TCGA database. The data which included somatic variants were reserved in the Mutation Annotation Format (MAF) form. 306 tumor samples were equally sectionalized into high- and low-immunity cohorts depending on the median level of ImmuneScore. DMGs were identified by comparing the high- and the low-immunity cohorts using R package maftools (Mayakonda et al., 2018), and *p* < 0.05 served as the significant threshold.

### Identification of DEGs in High- and Low-Immunity Cohorts

Differentially expressed genes were similarly identified by comparing between the high- and the low-immunity samples by differentiation analysis using package limma. DEGs that met the following criteria were considered significant: I. false discovery rate (FDR) <0.05; II. absolute value of log2 fold change (FC) > 1 (high-immunity cohort/low-immunity cohort).

### Gene Ontology (GO) and Kyoto Encyclopedia of Genes and Genomes (KEGG) Enrichment Analysis and Heatmaps of DEGs

Gene ontology and Kyoto encyclopedia of genes and genomes enrichment analyses of 1067 DEGs were conducted using R packages clusterProfiler, enrichplot, and ggplot2 to explore the biological functions and signaling pathways. The significantly enriched terms should be up to the following standards simultaneously: I. *p*-value < 0.05; II. *q*-value < 0.05. Heatmaps of DEGs were drawn using pheatmap package.

### Gene Set Enrichment Analysis

C2. CP. KEGG.v7.2 gene sets and Hallmark collections were acquired from Molecular Signatures Database (MSigDB), which were analyzed by GSEA via the GSEA software (version: 4.0.3). The significant gene sets were up to following standards: I. NOM *p*-value < 0.05; II. FDR *q*-value < 0.25.

### Analysis of TICs

CIBERSORT was utilized to approximately evaluate the proportion of TICs profile in the whole CC cases. Only cases with *p*-value < 0.05 were picked out for the follow-up analyses.

## Results

### Analysis Workflow of the Study

**FIGURE 1:**
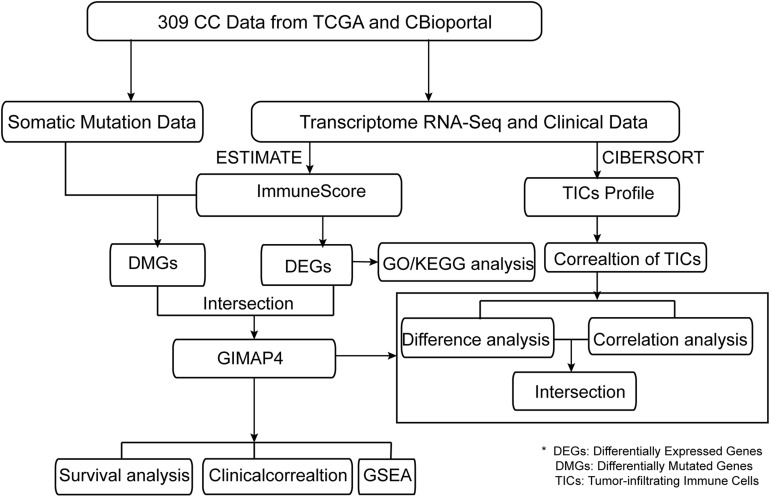
The analysis workflow of this study.

The presented study was carried out by the following analysis process (Figure 1). We employed CIBERSORT and ESTIMATE algorithms to separately calculate the ratio of TICs and the proportion of immune and stromal components in 309 CC cases after downloading the RNA-seq profiles from the TCGA database and the corresponding clinical information from cBioportal. Simultaneously, somatic mutation data was downloaded to identify the DMGs between high- and low-immunity cohorts according to the median ImmuneScore. Besides, DEGs were also identified based on the median ImmuneScore and we further conducted GO and KEGG analyses on these genes. Then, GIMAP4, HLA-B, and MAP2 were obtained using intersection analysis of DEGs and DMGs by ImmuneScore. We concentrated on GIMAP4 for further analyses, including correlation analysis of OS and clinicopathological characteristics, GSEA, correlation with TICs, etc.

### Characteristics of CC Patients From TCGA and CBioportal

We downloaded the RNA-seq expression data and corresponding clinical information for 309 CC cases from the TCGA and cBioportal. Then, 232 CC patients met the defined criteria, whose clinicopathological characteristics were listed in Table 1.

**TABLE 1:** Clinicopathological characteristics statistics of CC patients.

**Clinical characteristics**		**TCGA datasets (*n* = 232)**	
		***n***	**%**
Age	>50	84	36.2
	≤50	148	63.8
Stage	I	131	56.5
	II	54	23.3
	III	29	12.5
	IV	18	7.8
T classification	T1	126	54.3
	T2	65	28.0
	T3	17	7.3
	T4	10	4.3
	TX	14	6.0
N classification	N0	118	50.9
	N1	51	22.0
	NX	63	27.2
M classification	M0	101	43.5
	M1	10	4.3
	MX	121	52.2
OS times (months)	<12	42	18.1
	≥12	190	81.9

### Identification of DEGs in CC Patients

#### Scores Were Connected With the Prognosis of CC Patients

**FIGURE 2:**
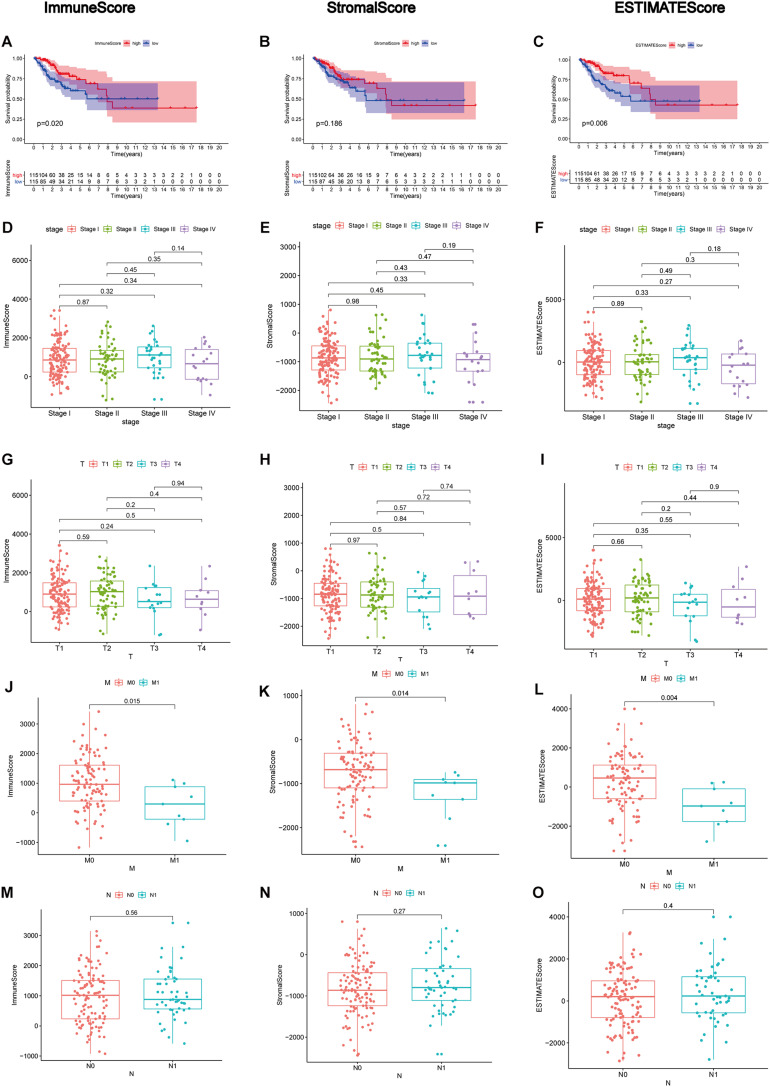
Correlation analyses of scores with survival and clinicopathological characteristics of CC patients. **(A–C)** Kaplan–Meier survival analysis for CC patients grouped into high or low scores in ImmuneScore, StromalScore, and ESTIMATEScore determined by comparing them with the median. *p* = 0.020, 0.186, and 0.006, respectively, by log-rank test. **(D–F)** Distribution of ImmuneScore, StromalScore, and ESTIMATEScore in the stage classification. The *p* = 0.45, 0.43, and 0.49, respectively, by Kruskal–Wallis rank sum test. **(G–I)** Distribution of three kinds of scores in the T classification (*p* = 0.5, 0.84, 0.55 by Kruskal–Wallis rank sum test for ImmuneScore, StromalScore, and ESTIMATEScore, respectively). **(J–L)** Distribution of scores in the M classification (*p* = 0.015, 0.014, 0.004 by Wilcoxon rank sum test for ImmuneScore, StromalScore, and ESTIMATEScore separately). **(M–O)** Distribution of scores in N classification. Similar to the preceding, *p* = 0.56, 0.27, 0.40, respectively, with Wilcoxon rank sum test.

After generating ImmuneScore, StromalScore, and ESTIMATEScore, we utilized the Kaplan–Meier survival analysis for these three scores, respectively. A higher ImmuneScore and StromalScore were represented for a greater proportion of the immune and stromal ingredients. ESTIMATEScore was reported to serve as the summation of ImmuneScore and StromalScore, meaning the tumor purity. The results revealed the correlation of the immune and stromal proportion with OS, indicating that ImmuneScore and ESTIMATEScore were positively correlated with OS (Figures 2A,C), despite no significant association in StromalScore (Figure 2B). Consequently, these results demonstrated that the immune component was more likely to indicate the prognosis of CC patients. Then, the clinical information of CC cases was analyzed to find the correlation between these three scores with the clinicopathological characteristics (Figures 2D–O), showing that ImmuneScore, StromalScore, and ESTIMATEScore were notably declined along with the progression of M classification (Figures 2J–L, *p* = 0.015, 0.014, 0.004, respectively, by Wilcoxon rank sum test). These findings clarified that both the immune and stromal components played a crucial part in the progression of CC, especially invasion and metastasis.

#### The DEGs Were Identified by ImmuneScore

**FIGURE 3:**
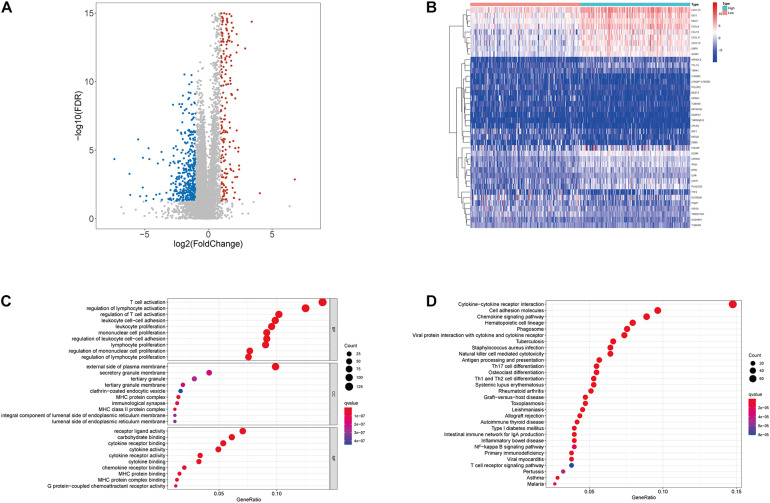
Volcano plot, Heatmap, and enrichment analysis of GO and KEGG for DEGs. **(A)** Volcano plot for DEGs. The blue and red dots represented the significantly downregulated and upregulated genes, respectively; and the gray dots represented the genes without differential expression. FDR < 0.05, | log2 FC | > 1 and *p* < 0.05 **(B)** Heatmap for DEGs generated by comparison of the high score group vs. the low score group in ImmuneScore. The row name of heatmap is the gene name, and the column name is the ID of samples which not shown in the plot. DEGs were determined by Wilcoxon rank sum test with FDR < 0.05 and | log2 FC | > 1 as the significance threshold. **(C,D)** GO and KEGG enrichment analysis for 1067 DEGs, terms with *p* and *q* < 0.05 were believed to be enriched significantly.

Since the immune component was more likely to forbade the prognosis of CC patients, we further conducted a comparison analysis between high- and low-immunity samples regarding the median level of ImmuneScore. A total of 1067 DEGs were obtained from ImmuneScore compared to the median, appearing up-regulation of 643 genes and down-regulation of 424 genes (Figure 3A). Then, the top 20 genes with up-regulation and down-regulation were respectively identified by the absolute values of log2 FC, and the heatmap was illustrated in Figure 3B. Moreover, GO analysis demonstrated that the DEGs were mainly linked to the immune functions, like T-cell activation and lymphocyte activation regulation (Figure 3C). Similarly, KEGG enrichment analysis results also revealed a high enrichment of immune-related biological processes, taking the cytokine–cytokine receptor interaction, cell adhesion molecules, and chemokine signaling pathway for instance (Figure 3D). Consequently, immune-related biological processes tended to represent DEGs’ main functions, demonstrating the immune component as an essential ingredient in the TME of CC patients.

### Identification of DMGs in CC Patients

**FIGURE 4:**
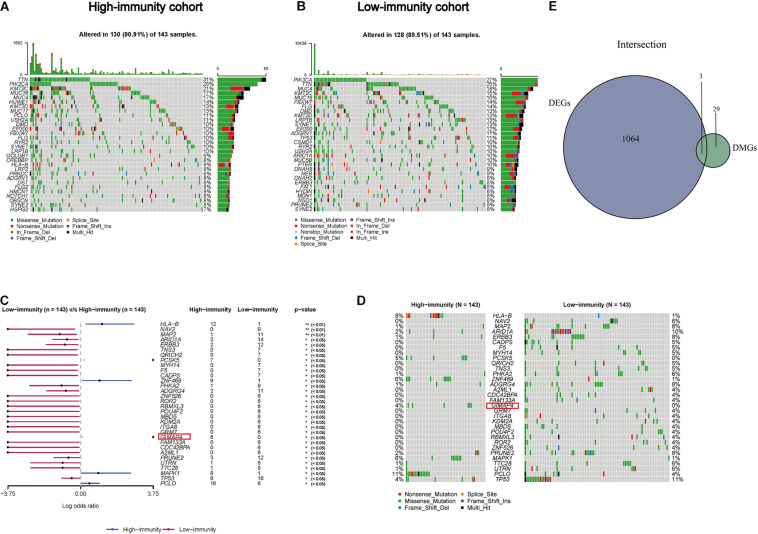
Somatic mutation analyses between high- and low-immunity groups and identification of common genes in DEGs and DMGs. **(A,B)** Waterfall plot shows the mutation distribution of the top 30 most frequently mutated genes. The central panel shows the types of mutations in each CC sample. The upper panel shows the mutation frequency of each CC sample. The bar plots on the right side show the frequency and mutation type of genes mutated in the high- and low-immunity cohort, respectively. The bottom panel is the legend for mutation types. **(C)** Forest plot displays the significant differentially mutated genes between two cohorts and GIMAP4 is marked out with a red rectangle. **p < 0.01, *p < 0.05. **(D)** Oncoplot shows the 32 DMGs between high- and low-immunity groups and GIAMP4 is marked out with a red rectangle. The central panel shows the types of mutations in each CC sample. The bottom panel is the legend for mutation types. **(E)** Venn plot showing the common factors of DEGs and DMGs.

Accumulating evidence has shown tumor-specific mutations could generate neoantigens, activate immunological recognition, and kill the tumor cells (Turajlic et al., 2017; Smith et al., 2019). To determine the correlation of gene mutation with the immune component in TME, we launched a further investigation to explore whether there existed differences in the genetic layer between the high- and low-immunity cohorts according to the median level of ImmuneScore. Somatic mutation data was analyzed and visualized in these two groups. The top 30 most frequently mutated genes of these two cohorts were displayed in Figures 4A,B. Interestingly, TTN, PIK3CA, MUC4, KMT2C, and MUC16 were the top mutations in both cohorts, which were reported to regulate various tumor biological processes in CC (Xu et al., 2017; Jiang et al., 2018; Shen et al., 2020), indicating that they are less participated in the process of immune infiltration but mainly involved in tumorigenesis and progression. Besides, there appeared a larger percentage of mutated genes in the high immunity group comparing to the low one, suggesting patients with more gene mutation tended to have higher immune infiltration. More interestingly, there were 32 DMGs between the two cohorts, ranked by order of *p*-value (Figures 4C,D and Supplementary Table 1). In addition, the following three factors, GIMAP4, HLA-B, and MAP2, were overlapped from the intersection analysis of DEGs and DMGs (Figure 4E). Among them, GIMAP4 of the high-immunity group expressed and mutated more by comparison with the low one, showing that the larger amount of GIMAP4 was likely to have more immune infiltration cells and thus enhanced the immunological responses of CC.

### GIMAP4 Expression Was Related to the Survival and Clinicopathological Characteristics in CC Patients

**FIGURE 5:**
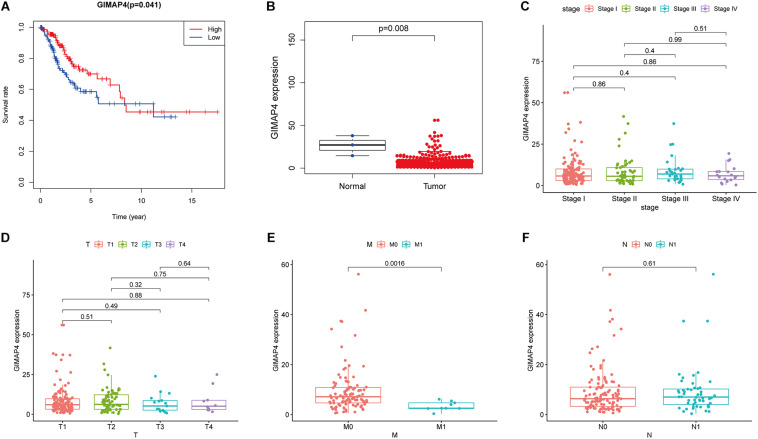
The differentiated expression of GIMAP4 in samples and correlation with survival and clinicopathological staging characteristics of CC patients. **(A)** Survival analysis for CC patients with different GIMAP4 expression. Patients were marked with high expression or low expression depending on comparing with the median expression level. *p* = 0.041 by log-rank test. **(B)** Differentiated expression of GIMAP4 in the normal and tumor sample. Analyses were conducted across all normal and tumor samples with *p* = 0.008 by Wilcoxon rank sum test. **(C–F)** The correlation of GIMAP4 expression with clinicopathological characteristics. Wilcoxon rank sum or Kruskal–Wallis rank sum test acted as the statistical significance test.

Th1/Th2 drifting effect was a common phenomenon in cancer development, under which circumstance the amount of Th2 was more massive than Th1, thus suppressing the anti-tumor immunity (Yang et al., 2010; Xu, 2014; Chen X. et al., 2019). GIMAP4 played an essential role in regulating lymphocyte apoptosis and was reported to be closely connected with the immune biological process of Th1/Th2 differentiation, and there appeared upregulation and downregulation of GIMAP4 under Th1- and Th2-promoting circumstance, respectively (Filen et al., 2009; Filen and Lahesmaa, 2010). Increasing evidence had revealed that GIMAP4 seemed to serve as a protective factor in several kinds of cancer, including lung cancer (Krucken et al., 2004; Lan et al., 2020). However, there were few available studies of GIMAP4 in CC until now. In our study, we divided all CC samples into GIMAP4 high- and low-expression groups on the basis of the GIMAP4 median expression. The phenomenon was observed using the survival analysis that CC patients in the GIMAP4 high expression group possessed a relatively longer OS than low expression (Figure 5A). Additionally, the further results indicated that the GIMAP4 expression in the normal cases was remarkably higher than the tumor samples by the Wilcoxon rank sum test (Figure 5B). After that, we conducted an analysis of GIMAP4 combined with clinicopathological characteristics, revealing that GIMAP4 expression decreased gradually following the advanced M classification (Figures 5C–F). The above comprehensive analyses brought out the results that GIMAP4 expression was a protective factor of CC patients, which was strongly associated with the prognosis, including survival and metastasis.

### GIMAP4 Might Serve as a Promising Indicator for Remodeling TME

**FIGURE 6:**
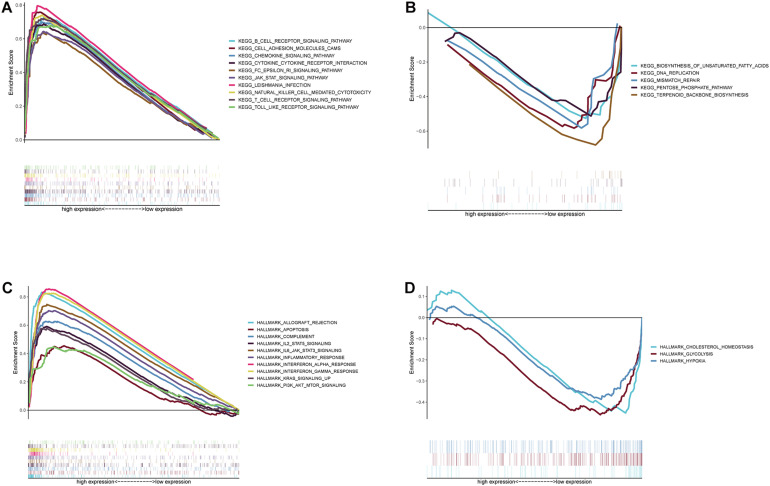
GSEA for samples with high GIMAP4 expression and low expression. **(A)** Enriched gene sets in C2 collection, the KEGG gene sets, by samples of high GIMAP4 expression. Each line is represented one particular gene set with unique color, and up-regulated genes are located on the left which approach the origin of the coordinates; by contrast, the down-regulated ones lay on the right of the *x*-axis. Only gene sets both with NOM *p* < 0.05 and FDR *q* < 0.25 were considered significant. Only several top gene sets are shown in the plot. **(B)** Enriched gene sets in C2 by the low BTK expression. **(C)** The enriched gene sets in HALLMARK collection by samples with high GIMAP4 expression sample. **(D)** The enriched gene sets in HALLMARK in the low GIMAP4 expression.

According to the above results, we finally concluded that GIMAP4 expression had a significant positive correlation with OS and clinicopathological characteristics, especially the M classification of CC patients. Besides, GSEA was ulteriorly conducted in the GIMAP4 high- and low-expression cohorts, respectively. On the one hand, for C2 collection defined by MSigDB, the GIMAP4 high-expression group genes had principal enrichment in immune biological processes, taking the B cell receptor signaling pathway, chemokine signaling pathway, and JAK-STAT signaling pathway for example (Figure 6A and Supplementary Table 2). Synchronously, the genes in the GIMAP4 low-expression cohort were mainly enriched in metabolic-related pathways, including biosynthesis of unsaturated fatty acids, terpenoid backbone biosynthesis, and pentose phosphate pathway (Figure 6B and Supplementary Table 2). On the other hand, similarly, multiple immune activities and metabolic functions were respectively enriched in the GIMAP4 high- and low-expression group for HALLMARK gene sets (Figures 6C,D and Supplementary Table 2). The above results illustrated that GIMAP4 might serve as a promising indicator for different TME status of CC.

### Relationship Between GIMAP4 With the Proportion of TICs

**FIGURE 7:**
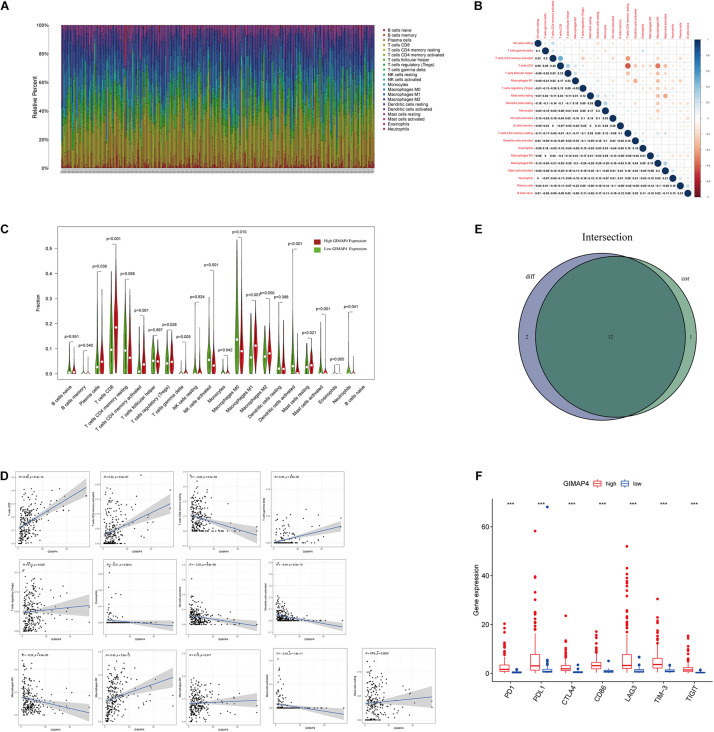
TIC profile in CC samples and correlation analysis, and correlation of TICs proportion and common ICPs with GIMAP4 expression. **(A)** Barplot shows the proportion of 21 types of TICs in CC tumor samples. The column names of the plot were sample ID. **(B)** Heatmap shows the correlation between 21 kinds of TICs and numeric in each tiny box, indicating the *p*-value of the correlation between two cells. The shadow of each tiny color box represented a corresponding correlation value between two cells, and the Pearson coefficient was used for the significance test. **(C)** Violin plot showed the ratio differentiation of 21 types of immune cells between CC tumor samples with low or high GIMAP4 expression relative to the median of GIMAP4 expression level, and Wilcoxon rank sum was applied for the significance test. **(D)** The Scatter plot showed the correlation of 13 kinds of TICs proportion with the GIMAP4 expression (*p* < 0.05). The blue line in each plot was a fitted linear model indicating the proportion tropism of the immune cell along with GIMAP4 expression, and the Pearson coefficient was used for the correlation test. **(E)** Venn plot displayed 12 kinds of TICs correlated with GIMAP4 expression codetermined by difference and correlation tests displayed in the violin and scatter plots, respectively. **(F)** The results showed that the expression of ICPs was significantly higher in the high GIMAP4 expression group than in the low one. ***p < 0.001.

We applied the CIBERSORT method to further confirm the relationship between GIMAP4 expression and the immune component, constructing 21 types of immune cell profiles in CC cases and analyzing the proportion of tumor-infiltrating immune subtypes (Figures 7A,B). Then, a total of 12 kinds of TICs were found to have a strong association with the GIMAP4 expression from the correlation and difference analyses (Figures 7C–E and Supplementary Table 3). The results revealed that seven TICs had a positive relationship with GIMAP4 expression, including macrophage M1, macrophage M2, CD8 + T cells, gamma delta T cells, CD4 + activated memory T cells, resting mast cells, and regulatory T cells; five kinds of TICs had a negative correlation with GIMAP4 expression, including macrophage M0, eosinophils, activated Dendritic cells, activated NK cells and activated mast cells. We could further confirm that GIMAP4 expression significantly influenced the immune activity in TME from the above results.

### Correlation of GIMAP4 With the Common ICPs

To evaluate the immunotherapy responses through GIMAP4 expression, we explored the correlation of GIMAP4 levels with common ICPs. The correlation between GIMAP4 expression and ICPs [programmed cell death 1 (PD1), programmed cell death-ligand 1 (PD-L1), cytotoxic T lymphocyte antigen 4 (CTLA4), T Cell Immunoglobulin Mucin 3 (TIM-3), lymphocyte activation gene-3 (LAG3), and T Cell Immunoreceptor With Ig And ITIM Domains (TIGIT), etc.] was performed, indicating the high expression of ICPs was observed in high GIMAP4 expression group (Figure 7F). The results demonstrated that patients with high GIMAP4 expression tended to have a better immunotherapy response because of the high levels of ICPs.

## Discussion

This study aimed to determine immune-related genes that were both differentially mutated and expressed in TME, which also conduced to the prognosis of CC patients, including OS and clinicopathological characteristics from the TCGA database and cBioportal. Then, a series of integrative bioinformatics analysis ultimately revealed that GIMAP4 met the above criteria, which was recognized to occupy an important position in immune-related biological functions and correlated with ICPs, demonstrating that GIMAP4 could serve as a promising indicator for remodeling TME and a potential predictor for prognosis and immunotherapy responses of CC patients.

Tumor microenvironment, especially the immune component, contributed a lot to the carcinogenesis and development of cancer. Transiting TME from tumor-friendly to tumor-suppressed was proved to be a beneficial strategy to improve the treatment of cancer (Duan et al., 2020; Zhang Z. et al., 2020). Therefore, it is urgently needed to determine the potential therapeutic targets that contribute to the above process. We came to a conclusion that the immune component in TME seemed to play a more critical part in the clinical outcomes of CC patients, including survival rate and M classification, illustrating that the immune component was closely related to the prognosis and the progression of CC, especially invasion and distant metastasis. Therefore, it is of great importance to investigate and clarify the interaction and cross-talk between immune infiltrating cells and tumor cells, thus developing a new idea for establishing much more effective therapeutic strategies to improve the CC treatments. Recently, many studies indicated that TICs, including T cells, macrophages, and natural killer cells, were promising prognostic biomarkers and had a tight connection to the development and prognosis of CC (De Nola et al., 2019; Wang et al., 2019; Zhang Y. et al., 2020). Besides, accumulating evidence has indicated that tumor-specific mutations could generate neoantigens, thus activating the immunological recognition and killing the tumor cells, indicating that modification of some specific genes could influence the status of TME (Turajlic et al., 2017; Smith et al., 2019). In addition, immunotherapy targeting ICPs had achieved tremendous success in multiple human cancers worldwide (Sun et al., 2020), including CC (Kagabu et al., 2020). However, the responses to immunotherapy using ICIs were relatively low in CC patients, and we could not ignore its immune-related adverse reactions (Frenel et al., 2017; Rischin et al., 2020). Therefore, we face a significant challenge to explore some novel candidates in TME to enhance the immunotherapy response of CC and decrease the immune-related adverse events. Moreover, most previously published studies focused only on the gene expression profiles or just on the somatic mutation data, which had limitations to reveal the potential mechanisms comprehensively in TME of CC. Hence, there is an urgent need for discovering the potential therapeutic targets using multi-layered data analysis. Here, we conducted an integrative bioinformatics analysis using transcriptomic RNA-seq data and somatic mutation data, revealing that the reduced GIMAP4 expression was significantly related to poor prognosis and advanced M classification. Meanwhile, our results also showed that GIMAP4 mutated more in the high-immunity group, confirming that its mutation could generate tumor-specific neoantigens and an activated immune system in CC. Further analysis of GSEA and CIBERSORT demonstrated that the high GIMAP4 expression group was in close connection with immune-related biological processes and activated immune infiltrating cells. Moreover, we also concluded that the expression of ICPs was higher in the GIMAP4 high expression group. Consequently, this present study indicated that GIMAP4 might be a potential prognostic signature for survival and immunotherapy response and an indicator for remodeling TME; most importantly, it is a therapeutic target for TME in CC.

The GIMAP genes are mapped on a chromosomal region within 7q35–7q36.1, coding for proteins primarily expressed in the immune system, which are closely correlated with the immune-related biological process such as Th cell differentiation, apoptosis of peripheral lymphocytes, and thymocyte development (Dion et al., 2005; Nitta et al., 2006; Filen et al., 2009). Additionally, except for their contribution to regulating the immune system, GIMAPs were also researched to serve as tumor suppressor genes, influencing the initiation and development of various cancers (Taniwaki et al., 2006; Shiao et al., 2008; Lan et al., 2020; Megarbane et al., 2020), whose expression was at deficient levels in various cancer tissues and cell lines (Krucken et al., 2004). GIMAP4 is the sole gene investigated to possess real GTPase activity among the GIMAP family (Heinonen et al., 2015), but its biological characteristics in CC are still far from clear. Our results illustrated that higher expression of GIMAP4 foreboded a better prognosis, and the expression of GIMAP4 was declining along with the advancing M classification of CC, indicating that GIMAP4 was a protective factor of CC patients in TME. Furthermore, GIMAP4 was known to have an apparent relationship with immune-related biological processes, taking the development and survival of T- and B-cell and apoptosis of T-cell for example (Heinonen et al., 2015). Various studies revealed that the immunobiological process of Th1/Th2 differentiation was vitally important in the development of tumor, during which process the amount of Th2 preponderated over Th1 in the advanced tumor patients, inhibiting the anti-tumor immunity (Neurath et al., 2002; Knutson and Disis, 2005). More critically, increasing evidence showed that GIMAP4 was closely connected with CD4 + Th lymphocytes differentiation by regulating the cytokines such as IL-12, IL-4, and INF-γ, which was up-regulated and down-regulated under Th1- and Th2-dominant circumstance, respectively (Filen et al., 2009; Filen and Lahesmaa, 2010; Heinonen et al., 2015). Thus, we concluded that GIMAP4 might reverse the Th1/Th2 drifting effect and increase the immunity of Th1, which might provide a new clue to enhance anti-tumor immunotherapy and improve treatment. However, the correlation of GIMAP4 with TME in CC remains unclear. Therefore, we further conducted GSEA to explore the relationship between GIMAP4 expression and TME, revealing that genes in GIMAP4 high-expression group had a high enrichment in immune biological processes, like chemokine signaling pathway, B cell receptor signaling pathway, and JAK-STAT signaling pathway. Interestingly, the GIMAP4 low-expression group genes were enriched most in metabolic-related activities, including biosynthesis of unsaturated fatty acids, terpenoid backbone biosynthesis, and pentose phosphate pathway. These results revealed that GIMAP4 might act as a satisfactory indicator for remodeling TME between immune and metabolism. Further CIBERSORT analysis for the ratio of TICs confirmed the effect of GIMAP4 on TME, especially the immune component, and the results revealed that CD8 + T cells and CD4 + activated memory T cells were positively correlated with GIMAP4 expression in CC patients. Besides, the expression of common ICPs was observed higher in the GIMAP4 high-expression group, demonstrating that GIMAP4 might have the potential to predict the immunotherapy responses in CC. Generally, GIMAP4 might act as an anti-tumor biomarker, a predictor for survival and immunotherapy response, and make contributions to representing the immune-dominant state in TME of CC according to the following facts that the upregulation of GIMAP4 along with the higher survival rate, early M classification of CC, the status conversion of TME from metabolism to immune, and the increment of anti-tumor TICs and ICPs.

Using integrated bioinformatics analysis, we identified GIMAP4 as an indicator for remodeling TME status, which could also serve as a promising predictor for clinical outcomes such as survival rate, distant metastasis, and immunotherapy response of CC. Therefore, further investigation should focus on clarifying the accuracy of an integrative analysis of GIMAP4 expression and confirming the specific correlation of GIMAP4 with Th1 and Th2, respectively. GIMAP4 also need to be tested in basic experiment and clinical trials.

## Conclusion

In this study, GIMAP4 was identified as a promising indicator for remodeling TME status for the first time by integrated bioinformatical analysis, which could also serve as a potential predictor for clinical outcomes such as overall survival rate, distant metastasis, and immunotherapy response of CC. Further studies were needed to explore the correlation of GIMAP4 with Th1/Th2 and to reveal the underlying mechanisms of the GIMAP4-related immunobiological process, which may improve the treatment of CC patients.

## Data Availability Statement

The datasets generated and analyzed in this study are available in the TCGA database (https://portal.gdc.cancer.gov) and cBioportal (http://www.cbioportal.org).

## Author Contributions

FX and JS conceived, designed, and wrote the manuscript. FX analyzed the data. SX helped with manuscript and data review. All authors read and approved the final manuscript.

## Conflict of Interest

The authors declare that the research was conducted in the absence of any commercial or financial relationships that could be construed as a potential conflict of interest.

## Footnotes

1 https://portal.gdc.cancer.gov/

2 http://www.cbioportal.org

## References

[B1] Cai N. Hu L. Xie Y. Gao J. H. Zhai W. Wang L. (2018). MiR-17-5p promotes cervical cancer cell proliferation and metastasis by targeting transforming growth factor-beta receptor 2. *Eur. Rev. Med. Pharmacol. Sci.* 22 1899–1906. 10.26355/eurrev_201804_14712 29687841

[B2] Cancer Genome Atlas Research Network Albert Einstein College of Medicine Analytical Biological Service Barretos Cancer Hospital Baylor College of Medicine Beckman Research Institute of City of Hope et al. (2017). Integrated genomic and molecular characterization of cervical cancer. *Nature* 543 378–384. 10.1038/nature21386 28112728 PMC5354998

[B3] Chen X. Zhang X. L. Zhang G. H. Gao Y. F. (2019). Artesunate promotes Th1 differentiation from CD4+ T cells to enhance cell apoptosis in ovarian cancer via miR-142. *Braz. J. Med. Biol. Res.* 52:e7992. 10.1590/1414-431X20197992 31038546 PMC6489539

[B4] Chen X. J. Wu S. Yan R. M. Fan L. S. Yu L. Zhang Y. M. (2019). The role of the hypoxia-Nrp-1 axis in the activation of M2-like tumor-associated macrophages in the tumor microenvironment of cervical cancer. *Mol. Carcinog.* 58 388–397. 10.1002/mc.22936 30362630

[B5] Chopra S. Gupta M. Mathew A. Mahantshetty U. Engineer R. Lavanya G. (2018). Locally advanced cervical cancer: a study of 5-year outcomes. *Indian J. Cancer* 55 45–49. 10.4103/ijc.IJC_428_17 30147092

[B6] Cohen P. A. Jhingran A. Oaknin A. Denny L. (2019). Cervical cancer. *Lancet* 393 169–182. 10.1016/S0140-6736(18)32470-X 30638582

[B7] De Jaeghere E. A. Laloo F. Lippens L. Van Bockstal M. De Man K. Naert E. (2020). Splenic (18)F-FDG uptake on baseline PET/CT is associated with oncological outcomes and tumor immune state in uterine cervical cancer. *Gynecol. Oncol.* 159 335–343. 10.1016/j.ygyno.2020.08.001 32859399

[B8] De Nola R. Menga A. Castegna A. Loizzi V. Ranieri G. Cicinelli E. (2019). The crowded crosstalk between cancer cells and stromal microenvironment in gynecological malignancies: biological pathways and therapeutic implication. *Int. J. Mol. Sci.* 20:2401. 10.3390/ijms20102401 31096567 PMC6567055

[B9] Di J. Rutherford S. Chu C. (2015). Review of the cervical cancer burden and population-based cervical cancer screening in China. *Asian Pac. J. Cancer Prev.* 16 7401–7407. 10.7314/apjcp.2015.16.17.7401 26625735

[B10] Dion C. Carter C. Hepburn L. Coadwell W. J. Morgan G. Graham M. (2005). Expression of the Ian family of putative GTPases during T cell development and description of an Ian with three sets of GTP/GDP-binding motifs. *Int. Immunol.* 17 1257–1268. 10.1093/intimm/dxh302 16103028

[B11] Duan Q. Zhang H. Zheng J. Zhang L. (2020). Turning cold into hot: firing up the tumor microenvironment. *Trends Cancer* 6 605–618. 10.1016/j.trecan.2020.02.022 32610070

[B12] Filen J. J. Filen S. Moulder R. Tuomela S. Ahlfors H. West A. (2009). Quantitative proteomics reveals GIMAP family proteins 1 and 4 to be differentially regulated during human T helper cell differentiation. *Mol. Cell Proteom.* 8 32–44. 10.1074/mcp.M800139-MCP200 18701445 PMC2621005

[B13] Filen S. Lahesmaa R. (2010). GIMAP Proteins in T-Lymphocytes. *J. Signal Transduct.* 2010:268589. 10.1155/2010/268589 21637352 PMC3100574

[B14] Frenel J. S. Le Tourneau C. O’Neil B. Ott P. A. Piha-Paul S. A. Gomez-Roca C. (2017). safety and efficacy of pembrolizumab in advanced, programmed death ligand 1-positive cervical cancer: results from the phase Ib KEYNOTE-028 trial. *J. Clin. Oncol.* 35 4035–4041. 10.1200/JCO.2017.74.5471 29095678

[B15] Heinonen M. T. Kanduri K. Lahdesmaki H. J. Lahesmaa R. Henttinen T. A. (2015). Tubulin- and actin-associating GIMAP4 is required for IFN-gamma secretion during Th cell differentiation. *Immunol. Cell Biol.* 93 158–166. 10.1038/icb.2014.86 25287446 PMC4355353

[B16] Herbst R. S. Baas P. Kim D. W. Felip E. Perez-Gracia J. L. Han J. Y. (2016). Pembrolizumab versus docetaxel for previously treated, PD-L1-positive, advanced non-small-cell lung cancer (KEYNOTE-010): a randomised controlled trial. *Lancet* 387 1540–1550. 10.1016/S0140-6736(15)01281-7 26712084

[B17] Hirose S. Murakami N. Takahashi K. Kuno I. Takayanagi D. Asami Y. (2020). Genomic alterations in STK11 can predict clinical outcomes in cervical cancer patients. *Gynecol. Oncol.* 156 203–210. 10.1016/j.ygyno.2019.10.022 31757465

[B18] Jiang W. He T. Liu S. Zheng Y. Xiang L. Pei X. (2018). The PIK3CA E542K and E545K mutations promote glycolysis and proliferation via induction of the beta-catenin/SIRT3 signaling pathway in cervical cancer. *J. Hematol. Oncol.* 11:139. 10.1186/s13045-018-0674-5 30547809 PMC6293652

[B19] Kagabu M. Nagasawa T. Sato C. Fukagawa Y. Kawamura H. Tomabechi H. (2020). Immunotherapy for uterine cervical cancer using checkpoint inhibitors: future directions. *Int. J. Mol. Sci.* 21:2335. 10.3390/ijms21072335 32230938 PMC7177858

[B20] Knutson K. L. Disis M. L. (2005). Tumor antigen-specific T helper cells in cancer immunity and immunotherapy. *Cancer Immunol. Immunother.* 54 721–728. 10.1007/s00262-004-0653-2 16010587 PMC11032889

[B21] Krishnan V. Schaar B. Tallapragada S. Dorigo O. (2018). Tumor associated macrophages in gynecologic cancers. *Gynecol. Oncol.* 149 205–213. 10.1016/j.ygyno.2018.01.014 29395307

[B22] Krucken J. Schroetel R. M. Muller I. U. Saidani N. Marinovski P. Benten W. P. (2004). Comparative analysis of the human gimap gene cluster encoding a novel GTPase family. *Gene* 341 291–304. 10.1016/j.gene.2004.07.005 15474311

[B23] Lan X. Lin W. Xu Y. Xu Y. Lv Z. Chen W. (2020). The detection and analysis of differential regulatory communities in lung cancer. *Genomics* 112 2535–2540. 10.1016/j.ygeno.2020.02.005 32045668

[B24] Langers I. Renoux V. Reschner A. Touze A. Coursaget P. Boniver J. (2014). Natural killer and dendritic cells collaborate in the immune response induced by the vaccine against uterine cervical cancer. *Eur. J. Immunol.* 44 3585–3595. 10.1002/eji.201444594 25229656

[B25] Mayakonda A. Lin D. C. Assenov Y. Plass C. Koeffler H. P. (2018). Maftools: efficient and comprehensive analysis of somatic variants in cancer. *Genome Res.* 28 1747–1756. 10.1101/gr.239244.118 30341162 PMC6211645

[B26] Megarbane A. Piquemal D. Rebillat A. S. Stora S. Pierrat F. Bruno R. (2020). Transcriptomic study in women with trisomy 21 identifies a possible role of the GTPases of the immunity-associated proteins (GIMAP) in the protection of breast cancer. *Sci. Rep.* 10:9447. 10.1038/s41598-020-66469-w 32523132 PMC7286899

[B27] Nero C. Ciccarone F. Pietragalla A. Scambia G. (2019). PTEN and gynecological cancers. *Cancers (Basel)* 11:1458. 10.3390/cancers11101458 31569439 PMC6826459

[B28] Neurath M. F. Finotto S. Glimcher L. H. (2002). The role of Th1/Th2 polarization in mucosal immunity. *Nat. Med.* 8 567–573. 10.1038/nm0602-567 12042806

[B29] Nitta T. Nasreen M. Seike T. Goji A. Ohigashi I. Miyazaki T. (2006). IAN family critically regulates survival and development of T lymphocytes. *PLoS Biol.* 4:e103. 10.1371/journal.pbio.0040103 16509771 PMC1393758

[B30] Ohno A. Iwata T. Katoh Y. Taniguchi S. Tanaka K. Nishio H. (2020). Tumor-infiltrating lymphocytes predict survival outcomes in patients with cervical cancer treated with concurrent chemoradiotherapy. *Gynecol. Oncol.* 159 329–334. 10.1016/j.ygyno.2020.07.106 32829964

[B31] Pesola F. Sasieni P. (2019). Impact of screening on cervical cancer incidence in England: a time trend analysis. *BMJ Open* 9:e026292. 10.1136/bmjopen-2018-026292 30679300 PMC6347909

[B32] Rischin D. Gil-Martin M. Gonzalez-Martin A. Brana I. Hou J. Y. Cho D. (2020). PD-1 blockade in recurrent or metastatic cervical cancer: data from cemiplimab phase I expansion cohorts and characterization of PD-L1 expression in cervical cancer. *Gynecol. Oncol.* 159 322–328. 10.1016/j.ygyno.2020.08.026 32917410

[B33] Schachter J. Ribas A. Long G. V. Arance A. Grob J. J. Mortier L. (2017). Pembrolizumab versus ipilimumab for advanced melanoma: final overall survival results of a multicentre, randomised, open-label phase 3 study (KEYNOTE-006). *Lancet* 390 1853–1862. 10.1016/S0140-6736(17)31601-X 28822576

[B34] Shen H. Guo M. Wang L. Cui X. (2020). MUC16 facilitates cervical cancer progression via JAK2/STAT3 phosphorylation-mediated cyclooxygenase-2 expression. *Genes Genom.* 42 127–133. 10.1007/s13258-019-00885-9 31736008

[B35] Shiao Y. M. Chang Y. H. Liu Y. M. Li J. C. Su J. S. Liu K. J. (2008). Dysregulation of GIMAP genes in non-small cell lung cancer. *Lung Cancer* 62 287–294. 10.1016/j.lungcan.2008.03.021 18462827

[B36] Smith C. C. Selitsky S. R. Chai S. Armistead P. M. Vincent B. G. Serody J. S. (2019). Alternative tumour-specific antigens. *Nat. Rev. Cancer* 19 465–478. 10.1038/s41568-019-0162-4 31278396 PMC6874891

[B37] Sun J. Zhang Z. Bao S. Yan C. Hou P. Wu N. (2020). Identification of tumor immune infiltration-associated lncRNAs for improving prognosis and immunotherapy response of patients with non-small cell lung cancer. *J. Immunother. Cancer* 8:e000110. 10.1136/jitc-2019-000110 32041817 PMC7057423

[B38] Taniwaki M. Daigo Y. Ishikawa N. Takano A. Tsunoda T. Yasui W. (2006). Gene expression profiles of small-cell lung cancers: molecular signatures of lung cancer. *Int. J. Oncol.* 29 567–575. 16865272

[B39] Turajlic S. Litchfield K. Xu H. Rosenthal R. McGranahan N. Reading J. L. (2017). Insertion-and-deletion-derived tumour-specific neoantigens and the immunogenic phenotype: a pan-cancer analysis. *Lancet Oncol.* 18 1009–1021. 10.1016/S1470-2045(17)30516-8 28694034

[B40] Wang J. Li Z. Gao A. Wen Q. Sun Y. (2019). The prognostic landscape of tumor-infiltrating immune cells in cervical cancer. *Biomed. Pharmacother.* 120:109444. 10.1016/j.biopha.2019.109444 31562978

[B41] Wei H. Wang X. W. Chen K. M. Ling S. R. Yi C. J. (2018). Analysis of gene mutation associated with tyrosine kinase inhibitor sensitivity of epidermal growth factor receptor in cervical cancer patients. *Eur. Rev. Med. Pharmacol. Sci.* 22 6280–6287. 10.26355/eurrev_201810_16036 30338793

[B42] Xu D. Liu S. Zhang L. Song L. (2017). MiR-211 inhibits invasion and epithelial-to-mesenchymal transition (EMT) of cervical cancer cells via targeting MUC4. *Biochem. Biophys. Res. Commun.* 485 556–562. 10.1016/j.bbrc.2016.12.020 27923652

[B43] Xu H. M. (2014). Th1 cytokine-based immunotherapy for cancer. *Hepatobiliary Pancreat. Dis. Int.* 13 482–494. 10.1016/s1499-3872(14)60305-2 25308358

[B44] Yang P. Qiu G. Wang S. Su Z. Chen J. Wang S. (2010). The mutations of Th1 cell-specific T-box transcription factor may be associated with a predominant Th2 phenotype in gastric cancers. *Int. J. Immunogenet.* 37 111–115. 10.1111/j.1744-313X.2010.00899.x 20193034

[B45] Yoshihara K. Shahmoradgoli M. Martinez E. Vegesna R. Kim H. Torres-Garcia W. (2013). Inferring tumour purity and stromal and immune cell admixture from expression data. *Nat. Commun.* 4:2612. 10.1038/ncomms3612 24113773 PMC3826632

[B46] Yuan Y. Cai X. Shen F. Ma F. (2020). HPV post-infection microenvironment and cervical cancer. *Cancer Lett.* 497 243–254. 10.1016/j.canlet.2020.10.034 33122098

[B47] Zhang Y. Li X. Zhang J. Liang H. (2020). Natural killer T cell cytotoxic activity in cervical cancer is facilitated by the LINC00240/microRNA-124-3p/STAT3/MICA axis. *Cancer Lett.* 474 63–73. 10.1016/j.canlet.2019.12.038 31904481

[B48] Zhang Z. Bao S. Yan C. Hou P. Zhou M. Sun J. (2020). Computational principles and practice for decoding immune contexture in the tumor microenvironment. *Brief Bioinform.* 1:12. 10.1093/bib/bbaa075 32496512

[B49] Zhao Z. Li J. Li H. Yuan Wu N. Y. Ou-Yang P. Liu S. (2020). Integrative bioinformatics approaches to screen potential prognostic immune-related genes and drugs in the cervical cancer microenvironment. *Front. Genet.* 11:727. 10.3389/fgene.2020.00727 32733542 PMC7359727

[B50] Zhou M. Zhang Z. Zhao H. Bao S. Cheng L. Sun J. (2018). An immune-related six-lncRNA signature to improve prognosis prediction of glioblastoma multiforme. *Mol. Neurobiol.* 55 3684–3697. 10.1007/s12035-017-0572-9 28527107

[B51] Zhou M. Zhao H. Xu W. Bao S. Cheng L. Sun J. (2017). Discovery and validation of immune-associated long non-coding RNA biomarkers associated with clinically molecular subtype and prognosis in diffuse large B cell lymphoma. *Mol. Cancer* 16:16. 10.1186/s12943-017-0580-4 28103885 PMC5248456

