# Supplementary material for: Integrated Bioinformatical Analysis Identifies GIMAP4 as an Immune-Related Prognostic Biomarker Associated With Remodeling in Cervical Cancer Tumor Microenvironment
Source: Front Cell Dev Biol. 2021 Jan 21;9:637400. doi: 10.3389/fcell.2021.637400 (PMC7858649; doi:10.3389/fcell.2021.637400)
Supplement: Supplementary file 1 [file Table_1.DOCX]

**Table S1. DMGs between high- and low-immunity groups**

| Hugo_ Symbol | High. immunity | Low. immunity | P. value | OR | CI. up | CI. low |
| --- | --- | --- | --- | --- | --- | --- |
| HLA-B | 12 | 1 | 0.003 | 12.922 | 558.432 | 1.865 |
| NAV2 | 0 | 9 | 0.003 | 0 | 0.488 | 0 |
| MAP2 | 1 | 11 | 0.005 | 0.085 | 0.600 | 0.002 |
| ARID1A | 3 | 14 | 0.010 | 0.198 | 0.734 | 0.036 |
| ERBB3 | 2 | 12 | 0.011 | 0.156 | 0.719 | 0.017 |
| CADPS | 0 | 7 | 0.014 | 0 | 0.676 | 0 |
| F5 | 0 | 7 | 0.014 | 0 | 0.676 | 0 |
| MYH14 | 0 | 7 | 0.014 | 0 | 0.676 | 0 |
| PCSK5 | 7 | 0 | 0.014 | Inf | Inf | 1.478 |
| QRICH2 | 0 | 7 | 0.014 | 0 | 0.676 | 0 |
| TNS3 | 0 | 7 | 0.014 | 0 | 0.676 | 0 |
| PHKA2 | 1 | 9 | 0.019 | 0.105 | 0.779 | 0.002 |
| ZNF469 | 9 | 1 | 0.019 | 9.481 | 420.091 | 1.284 |
| ADGRG4 | 2 | 11 | 0.020 | 0.171 | 0.806 | 0.018 |
| A2ML1 | 0 | 6 | 0.030 | 0 | 0.833 | 0 |
| CDC42BPA | 0 | 6 | 0.030 | 0 | 0.833 | 0 |
| FAM133A | 0 | 6 | 0.030 | 0 | 0.833 | 0 |
| GIMAP4 | 6 | 0 | 0.030 | Inf | Inf | 1.200 |
| GRM7 | 0 | 6 | 0.030 | 0 | 0.833 | 0 |
| ITGA8 | 0 | 6 | 0.030 | 0 | 0.833 | 0 |
| KDM2A | 0 | 6 | 0.030 | 0 | 0.833 | 0 |
| MBD5 | 0 | 6 | 0.030 | 0 | 0.833 | 0 |
| POU4F2 | 0 | 6 | 0.030 | 0 | 0.833 | 0 |
| RBMXL3 | 0 | 6 | 0.030 | 0 | 0.833 | 0 |
| ROR2 | 0 | 6 | 0.030 | 0 | 0.833 | 0 |
| ZNF526 | 0 | 6 | 0.030 | 0 | 0.833 | 0 |
| PRUNE2 | 3 | 12 | 0.031 | 0.235 | 0.897 | 0.042 |
| MAPK1 | 8 | 1 | 0.036 | 8.367 | 375.305 | 1.097 |
| TTC28 | 1 | 8 | 0.036 | 0.120 | 0.911 | 0.003 |
| UTRN | 1 | 8 | 0.036 | 0.120 | 0.911 | 0.003 |
| PCLO | 16 | 6 | 0.044 | 2.867 | 9.229 | 1.026 |
| TP53 | 6 | 16 | 0.044 | 0.349 | 0.975 | 0.108 |

**Table S2. Enriched gene sets**

| MSigDB collection | Gene set name | NES | NOM  p-val | FDR  q-val |
| --- | --- | --- | --- | --- |
| C2.cp.kegg.v7.2.symbols.gmt |  |  |  |  |
| GIMAP4 high expression | KEGG_CHEMOKINE_SIGNALING_PATHWAY | 2.717 | 0 | 0 |
|  | KEGG_NATURAL_KILLER_CELL_MEDIATED_CYTOTOXICITY | 2.681 | 0 | 0 |
|  | KEGG_CELL_ADHESION_MOLECULES_CAMS | 2.669 | 0 | 0 |
|  | KEGG_CYTOKINE_CYTOKINE_RECEPTOR_INTERACTION | 2.655 | 0 | 0 |
|  | KEGG_B_CELL_RECEPTOR_SIGNALING_PATHWAY | 2.618 | 0 | 0 |
|  | KEGG_T_CELL_RECEPTOR_SIGNALING_PATHWAY | 2.600 | 0 | 0 |
|  | KEGG_TOLL_LIKE_RECEPTOR_SIGNALING_PATHWAY | 2.600 | 0 | 0 |
|  | KEGG_LEISHMANIA_INFECTION | 2.583 | 0 | 0 |
|  | KEGG_JAK_STAT_SIGNALING_PATHWAY | 2.571 | 0 | 0 |
|  | KEGG_FC_EPSILON_RI_SIGNALING_PATHWAY | 2.523 | 0 | 0 |
|  |  |  |  |  |
| GIMAP4 low expression | KEGG_TERPENOID_BACKBONE_BIOSYNTHESIS | -1.909 | 0.004 | 0.144 |
|  | KEGG_BIOSYNTHESIS_OF_UNSATURATED_FATTY_ACIDS | -1.814 | 0.004 | 0.178 |
|  | KEGG_PENTOSE_PHOSPHATE_PATHWAY | -1.707 | 0.018 | 0.215 |
|  | KEGG_DNA_REPLICATION | -1.650 | 0.038 | 0.216 |
|  | KEGG_MISMATCH_REPAIR | -1.730 | 0.020 | 0.240 |
| h.all.v7.2.symbols.gmt |  |  |  |  |
| GIMAP4 high expression | HALLMARK_ALLOGRAFT_REJECTION | 2.712 | 0 | 0 |
|  | HALLMARK_IL2_STAT5_SIGNALING | 2.532 | 0 | 0 |
|  | HALLMARK_COMPLEMENT | 2.523 | 0 | 0 |
|  | HALLMARK_INFLAMMATORY_RESPONSE | 2.499 | 0 | 0 |
|  | HALLMARK_IL6_JAK_STAT3_SIGNALING | 2.498 | 0 | 0 |
|  | HALLMARK_INTERFERON_GAMMA_RESPONSE | 2.462 | 0 | 0 |
|  | HALLMARK_KRAS_SIGNALING_UP | 2.384 | 0 | 0 |
|  | HALLMARK_INTERFERON_ALPHA_RESPONSE | 2.214 | 0 | 0 |
|  | HALLMARK_APOPTOSIS | 2.007 | 0 | 0.005 |
|  | HALLMARK_PI3K_AKT_MTOR_SIGNALING | 1.917 | 0 | 0.009 |
|  |  |  |  |  |
| GIMAP4 low expression | HALLMARK_GLYCOLYSIS | -2.020 | 0 | 0.026 |
|  | HALLMARK_CHOLESTEROL_HOMEOSTASIS | -1.791 | 0.006 | 0.109 |
|  | HALLMARK_HYPOXIA | -1.599 | 0.026 | 0.191 |

**Table S3. TICs co-determined by difference test and correlation test**

| TICs | Correlation test (p-value) | Difference test (p-value) |
| --- | --- | --- |
| T cells CD8 | 0.46 (9.43E-14) | 3.53E-07 |
| T cells CD4 memory activated | 0.32 (6.48E-07) | 3.54E-04 |
| T cells regulatory (Tregs) | 0.14 (2.63E-02) | 2.80E-02 |
| T cells gamma delta | 0.26 (3.85E-05) | 5.33E-03 |
| NK cells activated | -0.28 (9.94E-06) | 2.77E-04 |
| Macrophages M0 | -0.26 (4.63E-05) | 1.03E-02 |
| Macrophages M1 | 0.43 (3.79E-12) | 6.66E-08 |
| Macrophages M2 | 0.15 (1.71E-02) | 4.97E-02 |
| Dendritic cells activated | -0.44 (6.91E-13) | 2.36E-08 |
| Mast cells resting | 0.19 (2.54E-03) | 2.11E-02 |
| Mast cells activated | -0.42 (1.85E-11) | 2.80E-08 |
| Eosinophils | -0.21 (1.38E-03) | 4.60E-03 |
